# Cattle immunized against the pathogenic l-α-glycerol-3-phosphate oxidase of *Mycoplasma mycoides* subs. *mycoides* fail to generate neutralizing antibodies and succumb to disease on challenge^☆^

**DOI:** 10.1016/j.vaccine.2013.08.100

**Published:** 2013-10-17

**Authors:** Musa M. Mulongo, Joachim Frey, Ken Smith, Christian Schnier, Hezron Wesonga, Jan Naessens, Declan McKeever

**Affiliations:** a International Livestock Research Institute, P.O. Box 30709, 00100 Nairobi, Kenya; b Royal Veterinary College, University of London, United Kingdom; c Institute of Veterinary Bacteriology, University of Bern, Bern, Switzerland; d Moredun Research Institute, Pentlands Science Park, Bush Loan, Penicuik, Midlothian, Scotland EH26 0PZ, UK; e National Veterinary Research Center, Muguga, P.O. Box 25, Kikuyu, Kenya

**Keywords:** Mycoplasma, Antibody, Vaccination, Cattle, Evolution, l-α-glycerol-3-phosphate oxidase

## Abstract

The membrane-associated enzyme l-α-glycerol-3-phosphate oxidase (GlpO) of *Mycoplasma mycoides* subs. *mycoides* (*Mmm*), the causal agent of contagious bovine pleuropneumonia (CBPP) has been identified as a virulence factor responsible for the release of toxic by-products such as H_2_O_2_ that mediate host cell injury. Since CBPP pathogenesis is based on host inflammatory reactions, we have determined the capacity of recombinant GlpO to generate *in vivo* protective responses against challenge in immunized cattle. We also investigated whether sera raised against recombinant GlpO in cattle and mice inhibit production of H_2_O_2_ by *Mmm*. Immunization of cattle with recombinant GlpO did not protect against challenge with a virulent strain of *Mmm*. Further, although both murine and bovine antisera raised against recombinant GlpO detected recombinant and native forms of GlpO in immunoblot assays with similar titres, only murine antibodies could neutralize GlpO enzymatic function. The data raise the possibility that *Mmm* has adapted to evade potential detrimental antibody responses in its definitive host.

## Highlights

• Cattle were vaccinated with *Mycoplasma mycoides mycoides* (*Mmm*) recombinant l-α-glycerol-3-phosphate oxidase.
• A mouse mAb to l-α-glycerol-3-phosphate oxidase was generated.
• Mouse mAb blocked H_2_O_2_ release by *Mmm*, but cattle antisera did not.
• Cattle were not protected after challenge.

## 1 Introduction

*Mycoplasma mycoides* subsp*. mycoides* (*Mmm*) is the causal agent of contagious bovine pleuropneumonia (CBPP), a serious respiratory disease of cattle that causes major economic losses, especially in sub-Saharan Africa [1,2]. In addition to tissue damage caused by *Mmm*, pathogenesis of CBPP arises partially from uncontrolled inflammatory responses [3]. The most widely deployed vaccine against CBPP is based on the live attenuated strain T1/44, which induces short-lived immunity and causes severe post-vaccinal lesions at the site of inoculation [4]. *Mmm* belongs to the class Mollicutes, which comprises the smallest, and simplest of the free-living, self-replicating bacteria [5,6]. Because of their limited genetic resources, Mollicutes are almost entirely dependent on host biosynthetic activity and metabolism for survival and attach to host cells for optimal growth [7].

Several authors demonstrated that *Mmm* translocates glycerol from host interstitial fluid through membrane-associated ATP-binding cassette transporter proteins GtsA, GtsB and GtsC. Once taken up, glycerol is phosphorylated to glycerol-3-phosphate (G3P) and subsequently oxidized to dihydroxyacetone phosphate, with the simultaneous release of H_2_O_2_ [8–10]. Pilo et al. identified the trans-membrane l-α-glycerol-3-phosphate oxidase (GlpO) as the enzyme responsible for oxidation of glycerol-3-phosphate and generation of H_2_O_2_ and proposed that the metabolite and other reactive oxygen species are translocated into the cytoplasm of in-contact cells, resulting in cellular damage [9]. Importantly, European strains of *Mmm* of the 1992–2000 epidemic, which predominantly caused chronic CBPP with less severe clinical signs, do not possess the *gtsB* and *gtsC* genes and, consequently, release lower amounts of H_2_O_2_ in the presence of physiological concentrations of glycerol [10]. The pathogenicity of H_2_O_2_ arising from glycerol metabolism by *Mmm* has been confirmed in an *in vitro* model using embryonic calf nasal epithelial (ECaNEp) cells [11]. Pretreatment of *Mmm* with antibody binding fragments (Fab) derived from rabbit polyclonal serum raised against rGlpO neutralizes enzyme activity, as shown by inhibition of H_2_O_2_ release in the presence of glycerol and abrogation of the cytotoxic effect on ECaNEp cells [9].

The *Mmm* vaccine strain T1/44 has an intact glycerol uptake and metabolic system [11], and it is possible that H_2_O_2_ contributes to post-vaccinal reactions observed at the site of inoculation. In addition, H_2_O_2_ may trigger the marked inflammation observed in the lungs of *Mmm* infected-cattle [9]. Therefore, a vaccine that targets GlpO and inhibits production of H_2_O_2_ by *Mmm* would be desirable, since it would be unlikely to elicit site reactions and would protect immune cattle undergoing infection from H_2_O_2_-associated cytotoxicity. Such a vaccine could be produced in sub-unit form using rGlpO or the T1/44 strain could be genetically modified to produce a mutant devoid of the active enzyme, but retaining GlpO epitopes capable of inducing antibodies with inhibitory capacity. The latter approach was used to target the metabolic enzyme dihydrolipoamide dehydrogenase of *Mycoplasma gallisepticum* and yielded a vaccine with protection superior to that of three other commercial vaccines [12]. The potential of this approach in respect of CBPP will depend on whether antibodies can be induced in cattle that bind GlpO and neutralize its activity, and whether such antibodies are protective. We have therefore evaluated the capacity of bovine and mouse GlpO antibodies to inhibit H_2_O_2_ release by *Mmm* using an *in vitro* assay of enzyme function. We have also investigated whether immunization of cattle with recombinant GlpO confers protection against challenge with live *Mmm*.

## 2 Materials and methods

### 2.1 Expression and purification of recombinant GlpO

The poly-histidine tailed full length 45-kDa recombinant GlpO protein was expressed and purified from transformed *E*. *coli* BL21 (DE3) harbouring *glpO* in the expression vector pETHIS-1 [13] and prepared as described by [9]. Briefly, transformed *E. coli* were grown and purified by Ni^2+^ chelation chromatography as described for other recombinant proteins [14]. Fractions were analyzed by 12.5% SDS-PAGE using standard protocols and purified GlpO was dialysed (28,000 kDa cut-off) against PBS (pH 7.4) and quantified using the Coomassie (Bradford) Protein Assay Kit (Pierce, Rockford, IL, USA) as described by the manufacturer.

### 2.2 *In vitro* culture and quantification of *Mmm*

Lyophilized aliquots of the virulent *Mmm* isolate B237, which originated from an acute case of CBPP in central Kenya [15] were reconstituted in pre-warmed (37 °C) Gourlay's medium [13] and propagated as described [16]. *Mmm* for challenge infection was quantified on the basis of colour changing units/ml (CCU/ml) using the method of Spearman Karber [17] as described [16]. *Mmm* was considered suitable for inoculation on the third day of passage if the culture appeared filamentous and was at pH 6.5.

### 2.3 Cattle immunization and experimental infection

Cattle immunization was conducted following guidelines of the U.K. Animals (Scientific) Procedures Act, 1986 and was approved by the ethical review committees of the National Veterinary Research Centre, Muguga (Kenya) and the Moredun Research Institute (UK). Fourteen yearling Zebu steers from a CBPP-free zone of Kenya and sero-negative by the CBPP complement fixation test (CFT) were randomly assigned into 2 groups each comprising 7 experimental animals and an equal number of controls. The experimental group was inoculated sub-cutaneously with 200 μg of rGlpO emulsified in 1 ml Complete Freund's adjuvant (CFA). Cattle were boosted after 4 weeks and again 6 weeks later with the same quantity of antigen emulsified in incomplete Freund's adjuvant (IFA; Sigma). The control group was similarly inoculated with CFA and boosted with IFA emulsions in PBS respectively.

Challenge was performed by endobronchial inoculation with 60 ml of culture containing ∼10^8^ CCU/ml of *Mmm* after sedation with 2 ml of 2% Xylazine (Bayer AG, Leverkusen, Germany). The inoculum was immediately followed by 30 ml of PBS and 15 ml of pre-warmed 10% low melting agar. Clinical signs were monitored daily and animals displaying continuous fever for ten days or recumbancy for 3 days were euthanized on ethical grounds. Surviving animals were euthanized at the end of the experiment on day 35. All animals were bled for preparation of sera by jugular venipuncture before challenge and prior to euthanasia.

### 2.4 Isolation of *Mmm* from experimental cattle

Lung tissue was immersed in 50 ml pre-warmed Gourlay's medium containing phenol red indicator and incubated at 37 °C in a humidified incubator with 5% CO_2_ for 3 days. Upon change of colour to yellow, cultures were streaked on tryptose agar plates (Difco), and incubated at 37 °C for 10 days. Four dilutions (1:10, 1:20, 1:30 and 1:40) of each broth culture were also prepared in Gourlay's medium. *Mmm* isolation was considered positive if all dilutions of broth cultures changed from orange colour to yellow within 14 days and corresponding agar plates showed mycoplasmal morphological features.

### 2.5 Mouse polyclonal and monoclonal antibodies

Polyclonal mono-specific serum against recombinant GlpO was obtained by intra-peritoneal immunization of Balb/C mice with 50 μg of purified rGlpO in 0.25 ml of PBS, emulsified in an equal volume of CFA, followed by two booster immunizations with the same quantity of antigen emulsified in IFA 2 and 6 weeks after primary immunization. Three weeks after antigen was detectable in immunoblots with serum dilutions of 1:1000 or more, mice received a final intravenous boost with 50 μg of rGlpO in 250 μl PBS without adjuvant. Mice were euthanized three days later and the spleens were harvested for production of monoclonal antibodies, while blood was collected by cardiac puncture for preparation of polyclonal sera. Monoclonal antibodies were prepared by fusion of splenocytes with X63.Ag8.653 myeloma cells as previously described [14]. Supernatants from wells containing hybridoma colonies were screened for recognition of rGlpO by immunoblot. Hybridomas harvested from positive wells were cloned and the selection procedure repeated. A hybridoma lines, designated MUG,1 was obtained and further tested for specificity.

### 2.6 Immunoblot

Immunobloting was performed as described [18], with some modifications. Briefly, proteins were electro-blotted on nitrocellulose (NC) membrane with pore size 0.45 μm (Protran Whatman, Gmbh Germany) under constant voltage of 20 V overnight at 4 °C. Membranes were blocked in 5% BSA in PBS-T (PBS 0.05% Tween 20) for 1 h then washed in PBS-T before being incubated for 1 h with the relevant primary antibody diluted in PBS-T. After a further wash, membranes were incubated for 1 h in horseradish peroxidase-conjugated goat anti-bovine IgG or goat anti-mouse IgG (Sigma) as appropriate. Bands were visualized by incubation in 30% DAB and 1% H_2_O_2_ in PBS.

### 2.7 Neutralization of enzyme activity by antibodies

The method of [10] was used to measure the ability of anti-GlpO sera to inhibit production of H_2_O_2_ by *Mmm* strain B237 *in vitro*. Thirty ml of *Mmm* culture in exponential growth phase (5 × 10^8^ cells/ml) was centrifuged at 8000 × *g* for 15 min. The supernatant was discarded and the pellet was washed by centrifugation using 10 ml sterile pre-warmed (37 °C) incubation buffer (0.1 M HEPES, 1.4 M NaCl, 0.1 M NaOH and 7 mM MgCl_2_; pH 7.3). The culture was re-suspended in incubation buffer to a volume consistent with an OD_650_ of 0.5–0.8 and 1 ml aliquots were dispensed in labelled 2 ml centrifuge tubes. After addition of 200 μl of diluted anti-GlpO serum, tubes were incubated for 1 h in a 37 °C water bath. Cells were then washed twice by centrifugation for 10 min at 8000 × *g* at 37 °C, resuspended in 2 ml incubation buffer containing glycerol at a final concentration of 100 μM and returned to the 37 °C water bath. Released H_2_O_2_ was quantified using Merckoquant Peroxidase test strips (Merck, Darmstadt, Germany). Starting 1 min after exposure to glycerol, a colorimetric strip was dipped in individual tubes for 1 s and the colour of the reaction zone was recorded. In this way, released H_2_O_2_ was measured at intervals of 5, 10, 15, 20 and 40 min. The experiment was repeated on three occasions and mean of each treatment was determined.

### 2.8 Bioinformatic analyses

Comparison of the GlpO protein sequence of *Mmm* (GenBank accession number CAE46341) with the EMBL/GenBank database was performed using the BLAST programme BLASTP [19]. Prediction of sub-cellular localizations was made with Psortb v3.0.2 (http://www.psort.org/psortb/; [20]). Repartition of detected proteins according to Cluster of Orthologous Group was performed with COGnitor (http://www.ncbi.nlm.nih.gov/COG/old/xognitor.html).

## 3 Results

### 3.1 Immunoblot

**Fig. 1:**
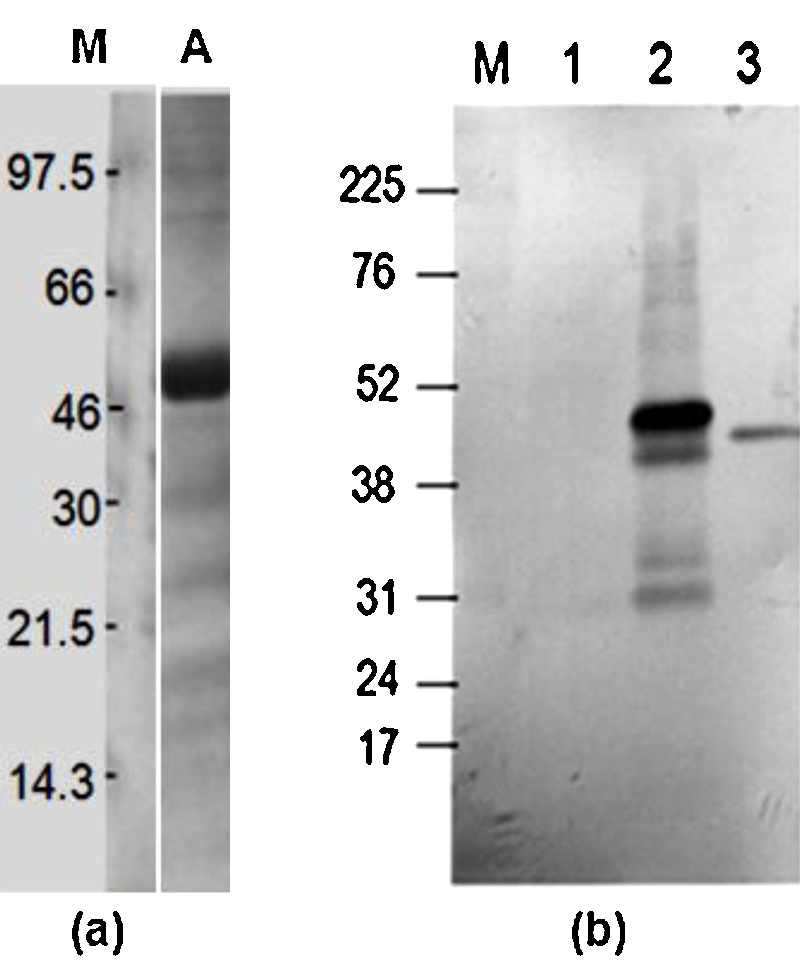
(a) Coomassie staining showing the 47.5 kDa rGlpO after purification by Ni^2+^ affinity chromatography and resolution on 12.5% SDS-PAGE. M: molecular weight. (b) Immunoblot detection of recombinant and native GlpO by mouse GlpO anti-serum; 5 μg samples of purified rLppQ-N′ (lane 1) purified rGlpO (lane 2) and 10 μg samples of total *Mmm* lysate (lane 3) were subjected to 12.5% SDS-PAGE, transferred to 0.45 μm nitrocellulose membrane then probed with mouse GlpO anti-serum (diluted at 1:1000). Rabbit anti-mouse IgG-HRP (diluted 1:4000) was used as the secondary antibody. Note, the small difference in size between recombinant GlpO (lane 2) and native GlpO as expressed by *Mmm* (lane 3) is due to the additional amino-terminal 10 histidine and carboxyterminal 6 histidine residues used for Ni^2+^ chelation chromatography standard.

**Fig. 2:**
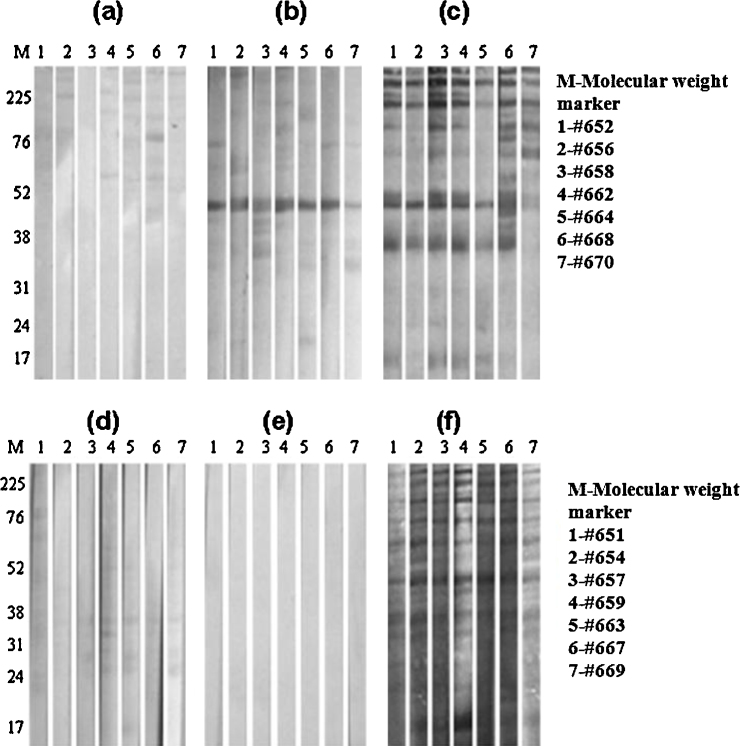
Detection of native GlpO by antisera from GlpO-immunized cattle by immunoblot. 10 μg samples of total *Mmm* lysate were subjected to 12.5% SDS-PAGE, transferred to 0.45 μm nitrocellulose membrane then probed with bovine GlpO anti-serum (diluted at 1:100); strips in panels (a)–(c) were respectively probed with pre-vaccination, pre-challenge and post-challenge sera from GlpO immunized cattle, while those in panels (d), (e) and (f) were probed with sera from non-immunized cattle collected at the same time-points. Goat anti-bovine IgG (H + L)-HRP (diluted 1:4000) was used as the secondary antibody.

SDS-PAGE analysis of elutes from the Ni-NTA matrix revealed the full-length 47 kDa rGlpO polypeptide along with additional minor bands of various sizes (Fig. 1a). A 100 ml-culture of *E. coli* yielded 2–4 mg of protein, of which rGlpO was the principal molecular component. Both mouse anti-GlpO serum (Fig. 1b) and serum prepared from GlpO-immunized cattle (Fig. 2b) reacted with native *Mmm* GlpO at a dilution of 1:100, although serum from animal #670 showed a comparatively weak signal. As expected, sera from cattle in the placebo group did not react with rGlpO (Fig. 2e). After challenge, in addition to antibody responses to other *Mmm* proteins, animals in both groups showed strong responses to native GlpO at a dilution of 1:100 (Fig 2c and d).

### 3.2 *In vitro* neutralization of *Mmm* H_2_O_2_ production by GlpO antisera

**Fig. 3:**
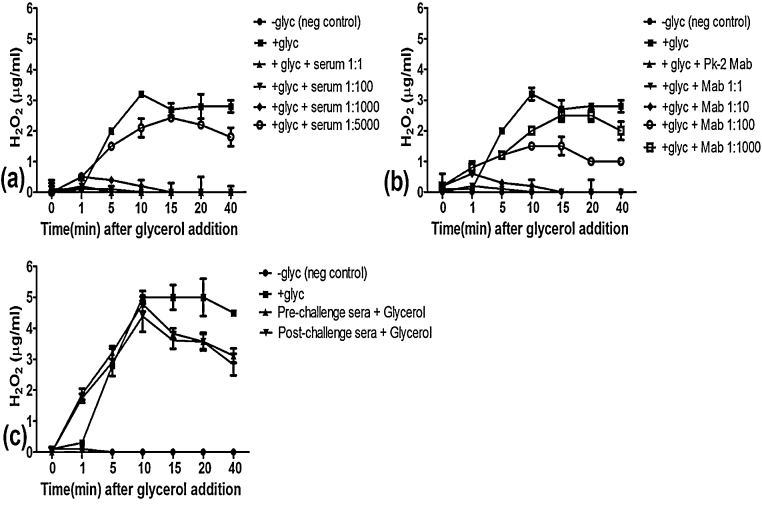
The capacity of anti-GlpO mouse polyclonal sera, MUG1 monoclonal antibodies and bovine sera to inhibit production of H_2_O_2_ by *Mmm*. *Mmm* were starved in PBS at 37 °C and simultaneously pre-incubated with serum or hybridoma supernatants before addition of 100 μM of glycerol and measurement of the quantity of H_2_O_2_ (*y*-axis) by *Mmm* at the intervals shown (a) Mouse polyclonal sera against GlpO; −glyc neg control: *Mmm* that did not receive glycerol after starvation; +glyc: *Mmm* that was not incubated with serum but received glycerol after starvation; +glyc+serum: *Mmm* incubated with serum at the dilution shown and also received glycerol after starvation. (b) Hybridoma supernatant containing anti-GlpO Mab: −glyc neg control: *Mmm* that did not receive glycerol after starvation; +glyc: *Mmm* that was not incubated with serum but received glycerol after starvation; +glyc+Pk2: *Mmm* that was incubated with hybridoma supernatant containing antibodies against a the *Mmm* capsular polysaccharide (Pk2) before addition of glycerol; +glyc+Mab: *Mmm* that was incubated with hybridoma supernatant containing Mabs against GlpO at the dilutions shown before addition of glycerol. (c) Bovine sera against GlpO (dilution 1:1): −glyc neg control: *Mmm* that did not receive glycerol after starvation; +glyc: *Mmm* that was not incubated with serum but received glycerol after starvation;+glyc+pre-challenge sera: *Mmm* that was incubated with sera from vaccinated but uninfected cattle before addition of glycerol; +glyc+post-challenge sera: *Mmm* that was incubated with vaccinated and infected cattle sera before addition of glycerol. For each data point, *n* = 7, and the error bars represent the means of standard deviation. (*x*-axis).

Release of H_2_O_2_ by untreated *Mmm* after addition of glycerol increased over 5–10 min to reach a plateau of 2–5 μg/ml, depending on the experiment (Fig. 3). Significant inhibition of peroxide release was observed in *Mmm* pretreated with varying dilutions of mouse anti-serum raised against full length rGlpO (Fig. 3a) before addition of glycerol, although inhibition was markedly reduced at a dilution of 1:5000. Release of H_2_O_2_ was not observed in control preparations consisting of *Mmm* and incubation buffer without glycerol.

Supernatant obtained from the MUG1 hybridoma (IgG2b) raised against rGlpO was also tested for its ability to inhibit H_2_O_2_ release by *Mmm* (Fig. 3b). This supernatant significantly blocked H_2_O_2_ release at a dilution of 1:10, while partial blocking was apparent at 1:100. However, the blocking capacity of this supernatant was minimal at 1:1000, with released H_2_O_2_ reaching near baseline levels after 15 min incubation. As expected, supernatants from mAb PK2, which binds *Mmm* membrane polysaccharide [21] did not inhibit H_2_O_2_ release.

Although seroconversion of cattle immunized with rGlpO was confirmed by immunoblot analysis, none of these sera inhibited H_2_O_2_ production by *Mmm*, before or after challenge infection (Fig. 3c). Similarly, sera derived from non-immunized cattle after challenge failed to inhibit H_2_O_2_ production by *Mmm* (data not shown).

### 3.3 Impact of GlpO immunization on the outcome of challenge

Clinical signs, antibody titres before euthanasia, survival, size of lung lesions and mycoplasma isolation are shown in Table 1. In both experimental and placebo groups, post-challenge clinical signs were consistent with acute CBPP (3 vaccinates and 1 placebo) and chronic CBPP (all remaining animals). Cattle displaying acute CBPP and were euthanized before the end point. While 6 vaccinates and 4 placebo animals developed fever (≥39.5 °C) within 2 weeks after challenge, no significant association was evident between immunization status and the likelihood of developing fever (Fisher's exact test, *p* = 0.55). Four vaccinates displayed CBPP lesions while only 1 placebo animal had a regressing lesion. Three immunized and five placebo animals had single or multiple sequestra (Table 1).

### 3.4 Bioinformatic analysis

**Fig. 4:**
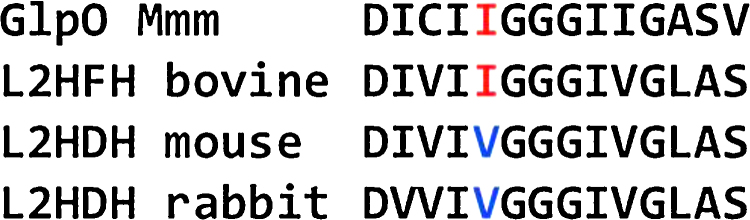
Sequence comparison of the FAD binding sites of the GlpO-related oxidases/dehydroxidases in *Mmm*, cattle, mice and rabbit. GlpO *Mmm*: Glycerolphosphate oxidase of *M. mycoides* subsp. *mycoides*; L2HFH: l-2-hydroxyglutarate dehydrogenase; L2HDH; l-2-hydroxyglutarate dehydrogenase. The comparison included in a first step the entire FAD binding site with 3 flanking a.a. The differences in the most conserved domain between GlpO *Mmm* and bovine L2HDH (I–I–G–G–G–I) with the corresponding domain of mouse and rabbit L2HDH are shown in colour.

Bioinformatic analysis revealed no GlpO homologue in cattle. The closest structurally related orthologue of GlpO of *Mmm* in cattle and other mammals is l-2-hydroxy glutarate dehydrogenase (L2HDH; Acc. no. BC151577), an enzyme belonging to the FAD-dependent superfamily of malate: quinone oxidoreductases of *Bos taurus* and *Bos grumiensis*, showing a maximum amino-acid (a.a.) identity of 26% over a coverage of 87% of the GlpO a.a. sequence, with an E-value of 2 × 10^−24^. The only similarity between these two orthologues was found within the FAD binding site. Comparing the a.a. sequence of the FAD binding site of *Mmm* GlpO with the corresponding sequences of L2HDH of cattle (*Bos taurus* L2HDH GenBank accession no. BC151577), mouse (*Mus musculus* L2HDH GenBank accession no. AK152450) and rabbit (*Oryctolagus cuniculus* L2HDH GenBank accession no. XM_002718232) reveals that the bovine L2HDH FAD binding site is most closely related to FAD of GlpO and to a lesser extent that of mouse, followed by that of rabbit (Fig. 4).

## 4 Discussion

We have evaluated the capacity of a recombinant form of *Mmm* GlpO to protect cattle against challenge with the organism. Using an immunization regimen based on Freund's adjuvant formulations, we found no evidence of protection in immunized animals, despite evident sero-conversion. Indeed, vaccinates seem to show increased pathological damage in comparison to challenge control cattle. This suggests either that GlpO is not a protective antigen, or that inappropriate immune responses were generated using Freund's adjuvant. No information is currently available on the nature of protective immunity in CBPP, although more favourable outcomes have been associated with *Mmm*-specific IFN-γ secreting CD4^+^ T cells [22,23] and, independently, with specific serum IgA responses [24]. We did not collect data on these parameters in our study.

With the exception of lung lesions in the immunized group and sequestration in the placebo group, no clear differences were apparent between the experimental and control groups with regard to post-challenge clinical and pathological parameters. However, immunized animals seemed to exhibit a more adverse outcome. Three of these animals were euthanized on humane grounds before the end of the experiment. Nonetheless, given the relatively low number of animals involved, no definitive conclusion is possible on whether immunization with recombinant GlpO results in a more severe outcome of disease following experimental infection. Enhanced pathology has been reported previously in cattle challenged with *Mmm* following immunization with a recombinant form of the LppQ protein of the organism formulated in ISCOMs [25]. The authors did not determine the underlying causes of the enhanced pathology and, given that a different adjuvant system was used, it is difficult to make direct comparisons between that study and our observations.

Immunoblot analysis confirmed that immunization with rGlpO induced detectable antibody responses against the native GlpO molecule, although these antibodies did not confer protection *in vivo*. Anti-GlpO polyclonal mouse serum and a mAb against rGlpO inhibited H_2_O_2_ *Mmm* production. This is consistent with previous reports, which demonstrated that sera from GlpO-immunized rabbits or mice could neutralize enzymatic activity of GlpO [9,26]. However, in our hands, sera from cattle immunized with rGlpO, or from cattle infected with CBPP, failed to inhibit H_2_O_2_ production by *Mmm*. In addressing this dichotomy, we have identified a single amino-acid substitution in the flavin-adenine-dinucleotide (FAD)-binding site (one of the main active sites of GlpO) of *Mmm* GlpO, as compared with the closest orthologue in cattle, mice and rabbits, L2HDH. It is tempting to speculate that adaptation of *Mmm* to mimic neutralizing epitopes of bovine orthologues is responsible for absence of neutralizing antibodies to GlpO of *Mmm* in cattle immunized with rGlpO. Such an eventuality might arise from a requirement to avoid immune recognition of neutralizing epitopes in cattle in order to retain enzyme function, in line with the importance of GlpO in its metabolism of glycerol [9].

In conclusion, our results indicate that induction of a humoral response to rGlpO of *Mmm* fails to protect cattle against challenge with the organism, with immunized animals showing apparently enhanced pathology. The evident lack of GlpO neutralizing activity in sera from immunized cattle when compared with that obtained from similarly immunized mice is intriguing, particularly in the light of a closer homology of GlpO with the bovine orthologue. Further work will be required to determine whether this truly reflects an adaptation of the organism to avoid the generation of a neutralizing response against the enzyme in cattle.

## Figures and Tables

**Table 1:** Summary of clinical, serological, pathological and bacteriological outcome of experimental challenge in GlpO vaccinated and placebo groups.

Animal ID	Respiratory distress	Fever	Survival (days)	Lung lesion (cm)	*Mmm* isolation
(a) GlpO vaccinated group					
652	Yes	Yes	35	13 × 11 6.5 × 3 (s)	+ve
656	No	Yes	35	9.3 × 8.5 (s)	+ve
658	Yes	No	35	No	−ve
662	No	Yes	15	10 × 19 12 × 12	+ve
664	No	Yes	35	10 × 7 (s) 4 × 2 (s)	+ve
668	No	Yes	20	15 × 22	+ve
670	No	Yes	15	26 × 15	+ve

(b) Placebo group					
651	Yes	No	35	12 × 11 (s)	+ve
654	No	No	35	1.8 × 0.8 (ms)	+ve
657	No	Yes	35	7.1 × 2 (s)	+ve
659	No	No	35	No	−ve
663	Yes	Yes	20	7 × 9 (rl)	+ve
667	Yes	Yes	35	10 × 9 (s)	+ve
669	No	Yes	35	4.8 × 4.3 (s)	+ve

The size shown is the longest dimensions (L × W) of the area covered by the lesion s, sequestrum; ms, multiple sequestra; rl, lesion in recession.

## Appendix A Supplementary data

The following KMZ files contain the Google maps of the most important areas described in this article.
